# Effects of a Movement-Based Mind-Body Intervention in Managing Osteoarthritis Symptoms in Older Adults

**DOI:** 10.1093/geroni/igab046.1767

**Published:** 2021-12-17

**Authors:** Juyoung Park, Carson Herron

**Affiliations:** 1 Florida Atlantic University, Boca Raton, Florida, United States; 2 Florida Atlantic University, Florida Atlantic University, Florida, United States

## Abstract

In a secondary analysis, this study examined differences in age (younger vs. older geriatric groups), gender, and living arrangement (living alone vs. living with others) in elderly patients with osteoarthritis (OA) who utilized chair yoga (CY) as an type of movement-based mind-body intervention (MMBI) for symptom management. A two-arm, assessor-blinded, randomized control trial was used to examine effects of CY (twice-weekly 45-minute sessions for 8 weeks) on pain interference, physical function, and psychosocial outcomes by gender, age, and living arrangement in older adults with OA who could not participate in traditional exercise. A total of 112 older adults completed CY or a health education program (HEP) and participated in five data collection points. Older women in the CY group showed greater reduction in pain interference during the CY intervention than those in HEP, F(4, 86) = 3.255, p = .016, η2 = .131. The younger group (ages 61 to 74) had decreased depression scores during the intervention, F(4, 87) = 2.598, p = .042, η2 = .107. Regardless of the intervention (CY or HEP), depression scores in older adults who were living alone decreased substantially during the intervention. Group-based and supervised CY interventions are recommended for older adults with OA to reduce pain interference, reduce depressive symptoms, and develop social networks. Online-based synchronous CY sessions may address physical activity needs and improve mental well-being in this population in light of physical distancing practices due to COVID-19.

